# Sleep disturbances and gastrointestinal dysfunction are associated with thalamic atrophy in Parkinson’s disease

**DOI:** 10.1186/s12868-019-0537-1

**Published:** 2019-10-22

**Authors:** Flavia Niccolini, Heather Wilson, Beniamino Giordano, Konstantinos Diamantopoulos, Gennaro Pagano, Kallol Ray Chaudhuri, Marios Politis

**Affiliations:** 1 Neurodegeneration Imaging Group, Institute of Psychiatry, Psychology and Neuroscience (IoPPN), King’s College London, London, UK; 2 National Parkinson Foundation International Centre of Excellence, Department of Basic & Clinical Neuroscience, King’s College London and Kings College Hospital, London, UK

**Keywords:** Parkinson’s disease, Non-motor symptoms, Cortical thickness, Thalamus, MRI

## Abstract

**Background:**

Non-motor symptoms are common aspects of Parkinson’s disease (PD) occurring even at the prodromal stage of the disease and greatly affecting the quality of life. Here, we investigated whether non-motor symptoms burden was associated with cortical thickness and subcortical nuclei volume in PD patients.

**Methods:**

We studied 41 non-demented PD patients. Non-motor symptoms burden was assessed using the Non-Motor Symptoms Scale grading (NMSS). Cortical thickness and subcortical nuclei volume analyses were carried out using Free-Surfer. PD patients were divided into two groups according to the NMSS grading: mild to moderate (NMSS: 0–40) and severe (NMSS: ≥ 41) non-motor symptoms.

**Results:**

Thalamic atrophy was associated with higher NMSQ and NMSS total scores. The non-motor symptoms that drove this correlation were sleep/fatigue and gastrointestinal tract dysfunction. We also found that PD patients with severe non-motor symptoms had significant thalamic atrophy compared to the group with mild to moderate non-motor symptoms.

**Conclusions:**

Our findings show that greater non-motor symptom burden is associated with thalamic atrophy in PD. Thalamus plays an important role in processing sensory information including visceral afferent from the gastrointestinal tract and in regulating states of sleep and wakefulness.

## Background

Non-motor symptoms have been recognised as an important component of Parkinson’s disease (PD). They occur in almost all the PD patients [1–3] and can precede the onset of motor symptoms [4, 5]. Sleep disturbances, depression, gastrointestinal and urinary dysfunction, fatigue and cognitive impairment are often unresponsive to dopamine replacement therapy and greatly affect the quality of life of PD patients [6].

Structural Magnetic Resonance (MR) imaging studies have used T1-weighted MRI together with automated computerised methods such as voxel-based morphometry (VBM) and cortical thickness measures to assess the morphology of cortical gray matter. Both morphometric analysis methods have been widely used to investigate neuroanatomical correlates of neurological disorders.

Previous MR imaging studies have shown cortical and subcortical changes in PD patients with cognitive impairment [7–12]. Mood disorders were also associated with cortical and subcortical changes in PD patients [13, 14]. Surface based morphometric analysis showed that depressed PD patients had significant increased cortical thickness in the orbitofrontal regions and insula compared to non-depressed PD patients [14] and loos of volume of the left nucleus accumbens was found in PD patients with apathy [13]. Significant grey matter volume loss in the lingual gyrus and superior parietal lobe was found in PD patients with visual hallucinations compared to healthy controls and PD patients without visual hallucinations [15]. Rapid eye movement (REM) behavior disorders (RBD) in PD patients was associated to atrophy of the pontomesencephalic tegmentum, medullary reticular formation, hypothalamus, thalamus, putamen, amygdala and anterior cingulate cortex [16]. Additionally, other symptoms, which can occur as non-motor manifestations of PD were associated with cortical and subcortical volumetric changes. Voxel-based morphometric analysis showed that patients with idiopathic restless leg syndrome (RLS) had significant loss of grey matter in the bilateral primary somatosensory cortex, which additionally extended into left-sided primary motor areas [17]. Voxel-based morphometric analysis showed loss of volumes in the left occipital cortex and parahippocampal gyrus of patients with chronic fatigue syndrome [18]. Volumetric changes were also observed in the right insula of patients with neurocardiogenic syncope suggesting that insular dysfunction may play a role in the development of neurocardiovascular symptoms [19].

Functional MR imaging (fMRI) studies have also shown abnormal functional communication and activation between brain regions, which play a role in the pathophysiology of non-motor symptoms in PD [20]. Dysfunction of the posterior cingulate cortex during executive task was linked to cognitive impairment in PD [21]. A research study using resting state fMRI (rs-fMRI) showed that frontal, postcentral, and anterior cingulate cortex regions are involved in fatigue in PD patients [22]. Other functional imaging studies demonstrated the involvement of both dorsal and ventral visual pathways in the pathogenesis of visual hallucinations in PD, [23]. Functional MR imaging provides a powerful tool to understand human brain organization. However, fMRI is complicated by several factors such as the fact that functional coupling changes dynamically, suggesting that it is constrained by, but not fully dictated by, anatomic connectivity. Moreover, fMRI is sensitive to head motion and to differences in the mental states of participants during the scans. Morphological MR imaging studies might provide a more objective insight in the pathophysiology of PD non-motor symptoms.

Clinical assessment of the overall burden grading of non-motor symptoms using validated questionnaires and scales is now possible [24, 25], however, the neural substrate of non-motor symptoms burden remain unclear. Previous studies using neuroimaging techniques such as positron emission tomography (PET) and quantitative susceptibility mapping (QSM) MRI have shown lack of association between non-motor symptoms burden as measured by Non-motor Symptoms Scale for PD (NMSS) and dopaminergic deficits and iron accumulation [26, 27]. In this study, we aimed for the first time to investigate whether non-motor symptoms burden is associated with cortical and subcortical morphological changes in a cohort of non-demented PD patients.

## Materials and methods

### Participants and clinical characteristics

Forty-one patients with a diagnosis of idiopathic PD according to the Queen Square Brain Bank criteria were recruited from specialist Movement Disorders clinics at King’s College Hospital, London (Table 1). None of these patients fulfilled the diagnostic criteria of PD mild cognitive impairment or PD dementia [28, 29]. All participants screened successfully to undertake MRI scanning under scanning standard criteria (http://www.mrisafety.com) and had no history of other neurological or psychiatric disorders.

**Table 1:** Clinical characteristics of Parkinson’s disease patients

	PD patients
No (M, %)	41 (24, 58.5%)
Age (years; median ± SD)	65.0 ± 9.3
Disease duration^a^ (months; median ± SD)	84.0 ± 71.8
Daily LED (mg; median ± SD)	480.0 ± 957.7
H&Y (median ± SD)	2.0 ± 1.0
NMSS (median ± SD)	37.0 ± 31.4
NMSS Domain 2: sleep/fatigue (median ± SD)	4.0 ± 7.7
NMS Domain 6: gastrointestinal tract (median ± SD)	3.0 ± 6.0
NMSQ (median ± SD)	8.0 ± 5.9
MMSE (median ± SD)	30.0 ± 1.3
PDQ-39 [(median ± SD) ± SD]	27.5 ± 24.4

*H&Y* Hoein & Yahr, *LED* levodopa equivalent dose, *MMSE* mini mental status examination, *NMSS* Non-motor Symptoms Scale, *NMSQ* Non-motor Symptoms Questionnaire, *PDQ-39* 39-item Parkinson’s disease Questionnaire

^a^From time of first appearance of Parkinson’s disease motor symptoms

NMSS and the patient self-reported Non-motor Symptoms Questionnaire (NMSQ) were used to assess non-motor symptoms. The NMSS is a healthcare professional-completed scale measuring the severity (mild, moderate, and severe) and frequency (every day, several times a week, once a week, and rarely) of 30 different NMS divided into 9 distinct domains [25]. Sleep disturbances includes four questions that assess excessive daytime sleepiness, fatigue, insomnia and RLS thus providing a comprehensive measure of daytime and nocturnal sleep disturbances in PD.

Neuropsychiatric symptoms were assessed with the Beck Depression Inventory-II (BDI-II), and the Hamilton Depression Rating Scale (HDRS). The Mini Mental Status Examination (MMSE) was used to assess general cognitive status. Motor symptom burden was assessed with the Hoehn & Yahr (H&Y) staging and it was performed OFF medication after overnight withdrawal of patient’s dopaminergic medications. Quality of life was measured with the patient self-reported 39-item PD Questionnaire (PDQ-39). Daily dopaminergic medication dose (LED) was calculated with a formula based on the theoretical equivalence to levodopa [28].

PD patients were divided into two groups according to the non-motor symptoms burden as assessed by the NMSS [25]: (1) PD patients with mild to moderate (NMSS levels 0–2, NMSS scores: 0–40; No: 23) and (2) severe (NMSS levels 3 and 4, NMSS scores: ≥ 41; No: 18) non-motor symptoms burden (Table 2).

**Table 2:** Clinical characteristics of Parkinson’s disease patients with mild to moderate and severe non-motor symptoms burden

	PD mild to moderate NMS burden (NMSS = 0–40)	PD severe NMS burden (NMSS ≥ 41)
No (%)	23 (56.1%)	18 (43.9%)
Gender (M, %)	15 (65.2%)	9 (50%)
Age (years; median ± SD)	58.5 ± 8.9	70.3 ± 8.3*
Disease duration^a^ (months; median ± SD)	16.1 ± 60.4	108.0 ± 73.8*
Daily LED (mg; median ± SD)	250.3 ± 438.3	690.0 ± 1219.6*
H&Y OFF (median ± SD)	1.0 ± 0.8	2.3 ± 1.2*
MMSE (median ± SD)	30.0 ± 1.6	29.5 ± 1.3
PDQ-39 (median ± SD)	9.5 ± 16.1	47.0 ± 24.4***

*H&Y* Hoehn & Yahr, *LED* levodopa equivalent dose, *MMSE* mini mental status examination, *NMSS* Non-motor Symptoms Scale, *PDQ-39* 39-item Parkinson’s disease Questionnaire, *UPDRS-III* Unified Parkinson’s Disease Rating Scale part III

*P < 0.05; ***P < 0.001

^a^From time of first appearance of Parkinson’s disease motor symptoms

The study was approved by the institutional review boards and the Surrey Borders research ethics committee. Written informed consent was obtained from all study participants in accordance with the Declaration of Helsinki.

### Imaging data analysis

MRI scans were acquired with a 32-channel head coil on a Siemens Magneton Trio Trim syngo MR B17 (Erlangen, Germany) 3-Tesla MRI scanners. A T1-weighted three-dimensional magnetization-prepared rapid acquisition gradient echo [MPRAGE; time repetition (TR) = 2300 ms, time echo (TE) = 2.98 ms, flip angle of 9°, time to inversion (TI) = 900 ms, matrix = 240 × 256; voxel size = 1 mm] was acquired. MRI scans were performed OFF medication after overnight withdrawal of patient’s dopaminergic medications to avoid motion artefact induced by motor complications such as levodopa-induced dyskinesia.

#### FreeSurfer analysis

FreeSurfer’s image analysis suite (version 5.3.0 http://surfer.nmr.mgh.harvard.edu) was used to process individual MRI scans for deriving region-of-interest (ROI)-based cortical thickness and subcortical volume analysis. Briefly, the whole-brain T1-weighted images underwent a correction for intensity homogeneity, skull strip, and segmentation into grey and white matter with intensity gradient and connectivity among voxels [30]. Cortical thickness was measured as the distance from the grey/white matter boundary to the corresponding pial surface [31]. Reconstructed data sets were visually inspected for accuracy, and segmentation errors were corrected. Subcortical nuclei volumes were derived by automated procedures, which assign a neuroanatomical label to each voxel in an MRI volume based on probabilistic information automatically estimated from a manually labelled training set [32]. All individual volumes were normalized for intracranial volume (ICV) automatically generated by FreeSurfer [33]. Since no laterality was observed and for minimizing the number of comparisons, average hemispheric CTh and subcortical nuclei values were processed in the statistical analysis.

### Voxel-wise volumetric analysis

Voxel-wise volumetric analysis was carried out using SPM12 implemented in Matlab 2015a [34]. T1 weighted MR images were spatially normalized to the T1 MNI template. MRI images were smoothed by application of 8 mm full-width at half maximum Gaussian kernel. Z-score maps were derived on a voxel basis using the general linear model. Subgroup analysis was carried out between PD patients with mild to moderate vs. severe non-motor symptoms (Fig. 2a), PD patients with sleep/fatigue disturbances vs. those without sleep/fatigue (Fig. 2b) and PD patients with gastrointestinal tract dysfunction vs. those without gastrointestinal tract dysfunction (Fig. 2c). The threshold for statistical significance was set to *P *< 0.05 cluster-wise corrected.

### Statistical analysis

Statistical analysis and graph illustration were performed with SPSS (version 20 Chicago, Illinois, USA) and GraphPad Prism (version 6.0c) for MAC OS X, respectively. For all variables, variance homogeneity and Gaussianity were tested with Shapiro–Wilk test. A sample size of 40 PD patients was adequate to detect significant correlations between MRI values and clinical measures of 0.79 with 80% power using 1% type I error. We recruited 41 PD patients to account for an approximate 5–10% drop-out. We interrogated correlations between ROI-based FreeSurfer’s volume analysis and clinical data using Spearman’s *r* correlation coefficient and we applied the Benjamini–Hochberg correction. Multivariate analysis of variance (MANOVA) was used to assess morphological differences in cortical thickness and subcortical nuclei volumes derived by FreeSurfer’s analysis between PD patients with mild to moderate and severe non-motor symptoms burden. If the overall multivariate test was significant, *P* values for each variable were calculated following Benjamini–Hochberg multiple-comparisons test in order to reduce false discovery rate. We set the false discovery rate cut-off at 0.05. Subsequently, MANOVA was repeated adding disease duration and daily LED as covariate since both disease duration and levodopa treatment have been associated to a cortical thinning [8, 35, 36]. PD patients were then subdivided in two groups according to the presence of sleep disturbances (NMSS subdomain 2; 29 PD patients with sleep disturbances) and GI dysfunction (NMSS subdomain 6; 23 PD patients with GI dysfunction). All data are presented as mean ± SD, and the level of *α* was set for all comparisons at *P *< 0.05, Benjamini–Hochberg corrected. For voxel-wise statistics appropriately weighted contrasts were used to derive Z-scores on a voxel basis using the general linear model; threshold for statistical significant was set to *P *< 0.05.

## Results

**Fig. 1:**
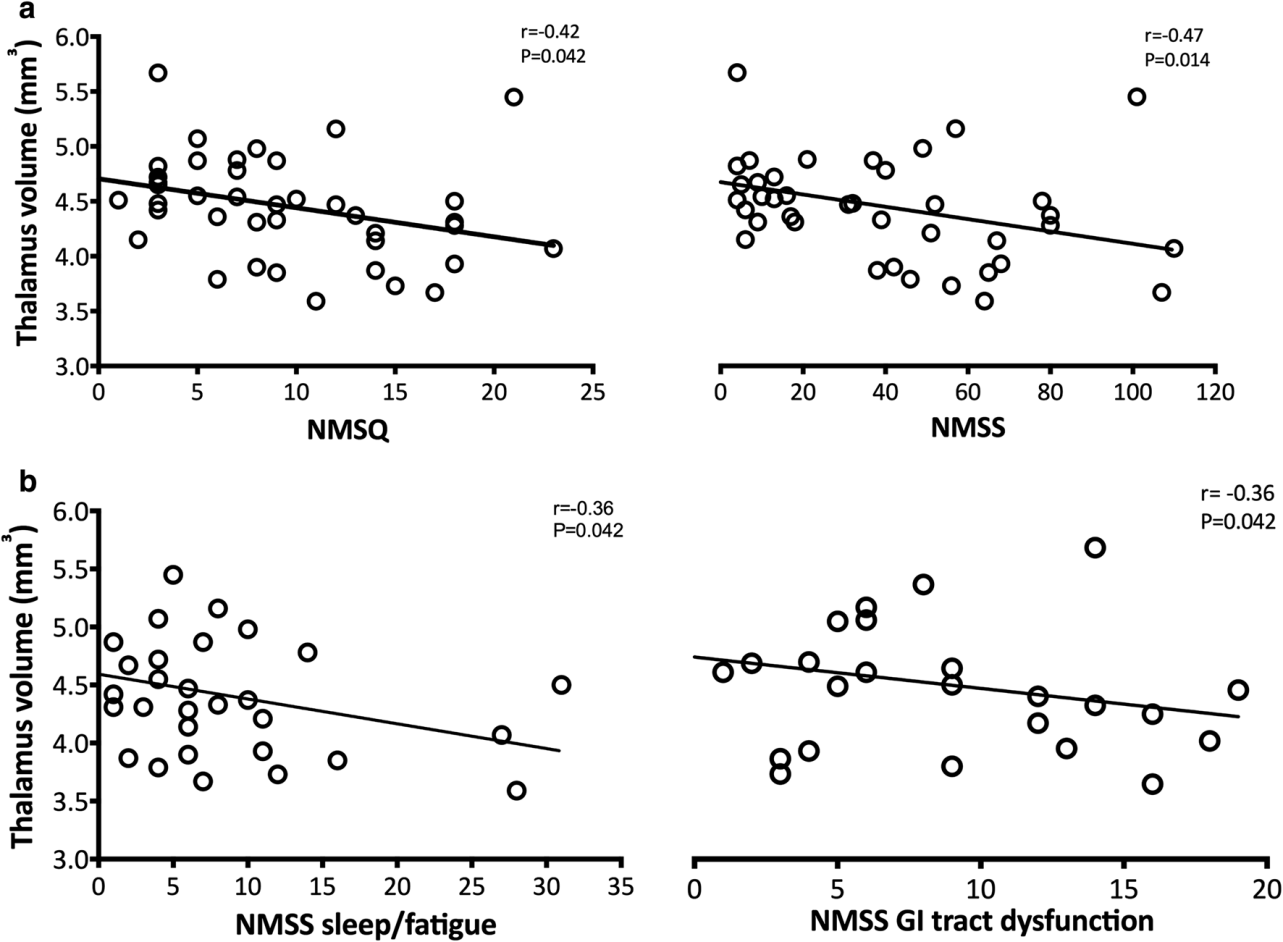
Correlations between thalamic atrophy and non-motor symptoms in Parkinson’s disease patients. Loss of thalamic volume correlated with **a** higher NMSQ (*r *= − 0.42, *P *= 0.042) and NMSS (*r *= − 0.47, *P *= 0.014) total scores; **b** higher NMSS domain 2 sleep/fatigue (*r *= − 0.36, *P *= 0.042) and NMSS domain 6 gastrointestinal tract dysfunction (*r *= − 0.36, *P *= 0.042) subscores

We found that loss of thalamic volume was associated to higher NMSQ (*r *= − 0.42, *P *= 0.042) and NMSS (*r *= − 0.47, *P *= 0.014) total scores (Fig. 1a). Within the NMSS subscores sleep/fatigue (*r *= − 0.36, *P *= 0.042) and gastrointestinal tract dysfunction (*r *= − 0.36, *P *= 0.042) were the non-motor symptoms that drove this correlation (Fig. 1b). No associations were found between non-motor symptoms burden and other subcortical nuclei volumes or cortical thickness (all *P *> 0.10).

### Structural changes in the group of PD patients with mild to moderate and severe non-motor disease burden

PD patients with severe non-motor symptoms burden were older (*P *= 0.024), had significant longer disease duration (*P *= 0.014), were receiving higher amounts of daily LED (*P *= 0.024) and had higher H&Y scores (*P *= 0.014) than those with mild to moderate non-motor symptoms burden (Table 2). PD patients with severe non-motor symptoms burden had also worse quality of life scores than those with mild to moderate non-motor disease burden (*P *< 0.001). PD patients with severe non-motor symptoms burden had higher NMSS Domain 2: Sleep/fatigue subscores compared to those with mild to moderate non-motor symptoms burden (8.68 ± 8.7 vs 2.17 ± 3.5; *P *< 0.001). In specific, PD patients with severe non-motor symptoms burden had higher scores at Subdomain 5: Does the patient have difficulties falling or staying asleep? compared to those with mild to moderate non-motor symptoms burden (2.42 ± 1.4 vs 0.87 ± 1.7; *P *< 0.01).

**Fig. 2:**
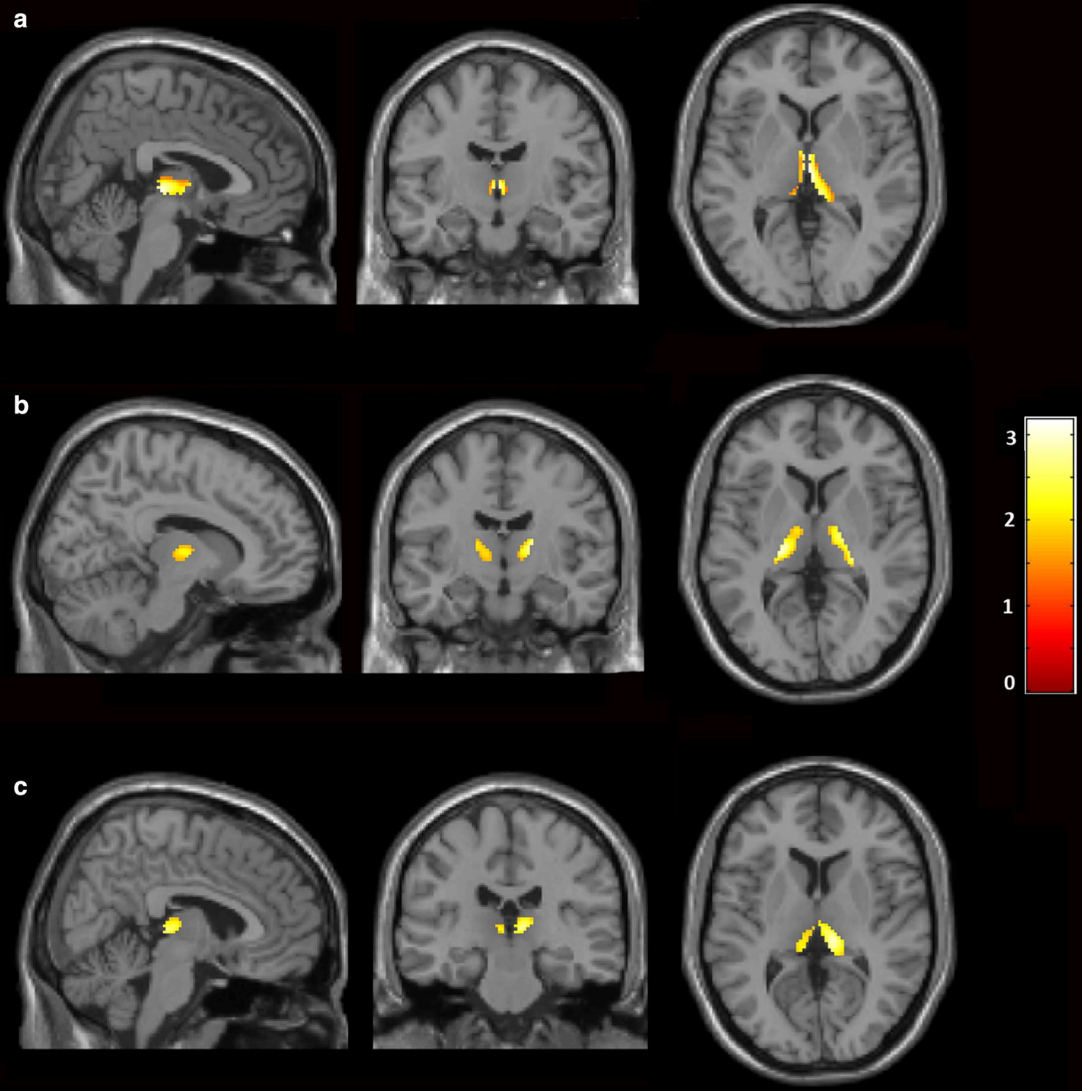
Thalamic atrophy in Parkinson’s disease patients. Statistical parametric maps of **a** PD patients with mild to moderate *vs.* severe non-motor symptoms; **b** PD patients with sleep/fatigue disturbances *vs.* those without sleep/fatigue; **c** PD patients with gastrointestinal tract dysfunction *vs.* those without gastrointestinal tract dysfunction. Yellow–red areas represent voxel clusters of significant volume loss. The colour stripe indicates z-values. MNI co-ordinates: **a** x = 3.85, y = − 12.23, z = 3.37; **b** x = 11.55, y = − 11.46, z = 4.90; **c** x = 9.24, y = − 26.85, z = 7.22

We found significant differences in subcortical nuclei volumes between the groups of PD patients with mild to moderate and severe non-motor symptoms (F (9,21) = 1.16; *P *= 0.045; Table 3). PD patients with severe non-motor symptoms burden had significant loss of thalamic volume compared to the group with mild to moderate non-motor symptoms burden (*P *= 0.048). There were no differences in caudate, putamen, globus pallidus, nucleus accumbens, amygdala and hippocampus between the two groups. Since PD patients with severe non-motor symptoms burden had longer disease duration, higher H&Y scores and were on higher daily LED, we repeated the multivariate analysis adding disease duration, H&Y and daily LED as covariate and this did not influence the results. Whole brain voxel-wise analysis between the group of PD patients with mild to moderate and severe non-motor symptoms confirmed the results from Freesurfer’s analysis. Whole brain analysis revealed clusters of significant volume loss in PD patients with severe non-motor symptoms in the thalamus (*P *< 0.05; Fig. 2a).

**Table 3:** Subcortical nuclei volume in Parkinson’s disease patients with mild to moderate and severe to very severe non-motor symptoms burden

	PD mild to moderate NMS burden (NMSS = 0–40)	PD severe NMS burden (NMSS ≥ 41)	*P* value*	% change
Caudate (median ± SD)	2.30 ± 0.2	2.40 ± 0.3	> 0.10	+ 2.5
Putamen (median ± SD)	3.30 ± 0.3	3.20 ± 0.3	> 0.10	− 3.6
Globus Pallidus (median ± SD)	1.00 ± 0.2	1.00 ± 0.1	> 0.10	− 9.1
Thalamus (median ± SD)	4.60 ± 0.4	4.20 ± 0.5	*0.048*	− *9.1*
Hippocampus (median ± SD)	2.50 ± 0.3	2.40 ± 0.3	> 0.10	− 7.7
Amygdala (median ± SD)	1.00 ± 0.1	0.96 ± 0.1	0.06	− 11.3
Nucleus accumbens (median ± SD)	0.30 ± 0.1	0.28 ± 0.1	0.06	− 9.1

Italic values indicate the significant result *P* < 0.05

* *P* values are Benjamini–Hochberg corrected for multiple comparisons

We then subdivided the PD patients into two groups according to the presence of sleep/fatigue disturbances and gastrointestinal tract dysfunction. PD patients with sleep/fatigue disturbances showed significant thalamic atrophy compared those without sleep/fatigue disturbances (*P *= 0.033; Fig. 2b). Significant thalamic volume loss was also observed in PD patients with gastrointestinal tract dysfunction compared those without gastrointestinal tract dysfunction (*P *= 0.043; Fig. 2c).

No significant differences in cortical thickness were found between the two groups (all *P *> 0.10). Vertex-wise analysis also did not show any significant differences in cortical areas between the PD patients with mild to moderate and severe non-motor symptoms burden.

## Discussion

Our findings indicate that higher non-motor symptoms burden is associated with thalamic atrophy in PD patients and in specific sleep/fatigue disorders and gastro-intestinal dysfunction were the non-motor symptoms, which drove this correlation. It is unclear why thalamic atrophy has been associated only to two non-motor symptoms. One possible explanation is that the thalamus plays a key role in modulating the flow of sensory and motor information to and from the cerebral cortex thus regulating the sleep/wake cycle pathway and gastrointestinal function.

While, morphological studies earlier have shown thalamic atrophy in PD patients, this is the first study showing a significant association between thalamic atrophy and non-motor symptoms. Non-motor symptom burden grading is currently the only validated clinical system to assess the overall impact of non-motor symptoms [25]. In clinical practice, this is important as many studies, which address non-motor symptoms may do so in a piecemeal fashion, addressing selected non-motor symptoms such as cognition rather than the overall burden. Studies have also shown that non-motor symptoms burden, rather than individual non-motor symptoms may be a key determinant of quality of life in PD [37]. Based on this classification system, when we divided our cohort of PD patients into two groups according to the severity of non-motor symptoms, we found that PD patients with severe non-motor symptoms burden had 9% significant loss of thalamic volume compared to those with mild to moderate non-motor-symptoms burden.

We found significant correlation between worse sleep/fatigue disturbances and thalamic atrophy in our cohort of PD patients. Sleep disturbances are one of the most common non-motor symptoms in PD occurring in up to 90% of PD patients [37–39]. Sleep disturbances may precede by many years the classic motor abnormalities of PD^33^ and their frequency increases with the progression of the disease [40, 41]. Sleep disturbances in PD can be divided in daytime manifestation, such as excessive daytime sleepiness, and nocturnal disturbances, which include insomnia, obstructive apnoea, restless legs syndrome (RLS) and rapid eye movement (REM) sleep behaviour disorder (RBD) [37].

The thalamus modulates the flow of sensory and motor information to and from the cerebral cortex and plays an important role in regulating the sleep/wake cycle pathway [42]. In PD, loss of dopamine in mesocorticolimbic pathway targeting the thalamus causes sleep/wake cycle dysfunction leading to disorders of thalamocortical arousal with lack of normal thalamocortical rhythms during night-time and excessive sleepiness during daytime [43, 44]. The role of thalamus in sleep disturbances is supported by preclinical and clinical data that showed lesions of the thalamus in cats and bilateral thalamic lesions in humans cause severe and persistent insomnia [45, 46]. Moreover, neuron loss was observed in the thalamus of patients with familial and sporadic fatal insomnia [47, 48]. Liu et al. [48] have shown loss of regional grey matter in the bilateral thalamus of 12 healthy subjects following 72 h sleep deprivation suggesting a close link between sleep disturbance and thalamus volume. Resting state functional MR imaging studies have shown reduced spontaneous activity in the thalamus of subjects with insomnia, which was associated with early morning awakening suggesting that hypoactivity of the thalamus may lead to an increased transmission of sensory signals from the ascending reticular activating system which cause awakening [48]. Thalamocortical functional connectivity was also decreased in 14 healthy volunteers following 36 h of sleep deprivation [49] and impaired thalamocortical connectivity is also involved in RLS symptoms [50, 51]. Thus, thalamic atrophy may account for the sleep/disturbances observed in PD.

Thalamic atrophy was also associated with worse gastrointestinal symptoms in our cohort of PD patients. The gastrointestinal tract is affected earlier in the course of PD and could play an important role in the pathogenesis of the disease providing a site of initiation for the accumulation and propagation of α-synuclein [52]. Gastrointestinal dysfunction in PD encompasses several symptoms ranging from oral issues such as drooling and swallowing difficulties to gastroparesis and constipation [53]. The thalamus receives signals from the gastrointestinal tract through the spinal or vagal afferents and projects to cortical areas, which process visceral sensation [54]. Previous MR imaging studies have shown decreased grey matter density in the thalamus of patients with irritable bowel symptoms [55, 56]. Moreover, white matter changes encompassing lower fractional anisotropy and increased mean diffusivity were observed in the basal ganglia and thalamus of patients with irritable bowel symptoms suggesting that patients with chronic gastrointestinal disorders may display microstructural changes within the cortico-basal ganglia-thalamic loop [57]. One of the symptoms assessed by the NMSS domain 6 is the difficulty in swallowing, functional MR imaging has shown that the thalamus, specifically the ventral posterior lateral nucleus, is involved in the oral-sensory stimulation and cerebral control of swallowing [58]. Our findings suggest that thalamus may play a role in the development of gastrointestinal dysfunction in PD.

Although PD patients with severe non-motor symptoms had longer disease duration, higher H&Y scores and were on higher daily LED, we did not find any effect of disease severity and dopaminergic treatment on the association between non-motor symptoms and thalamic atrophy. Whereas cortical grey matter atrophy has been shown to be related to disease duration, basal ganglia and in specific striatal atrophy was not correlated with duration of the disease [59]. Moreover, a recent study investigating non-motor symptoms burden in a large cohort of PD patients has shown high prevalence of non-motor symptoms even at the early-untreated stage of the disease, thus suggesting that the non-motor symptoms burden is independent from disease severity or dopaminergic treatment [60]. PD patients with severe non-motor symptoms had also worse quality of life scores than those with mild to moderate non-motor symptoms. This is in line with previous studies showing that non-motor symptoms have a greater impact on quality of life than motor symptoms and is common even at the early stage of the disease [61, 62].

A limitation of this study is the lack of an age-sex matched control group, which could have clarified whether thalamic atrophy is a distinct feature of PD unrelated to non-motor symptoms burden. Previous morphological studies using a control group have shown thalamic atrophy in PD patients with and without mild cognitive impairment, which was associated to worse cognitive impairment at 18-month follow-up [63].

## Conclusions

This is the first study showing an association between higher non-motor symptom burden and thalamic atrophy in PD. Among the non-motor symptoms, sleep/fatigue disturbances and gastrointestinal dysfunction were the non-motor symptoms that drove this correlation. Further studies, investigating morphological brain changes and non-motor symptoms burden in a larger cohort of PD patients and using specific scale to assess night and day time sleep disturbances such as the PD Sleep Scale and Epworth Sleep Scale and instrumental assessments for GI dysfunction such as endoscopic evaluation of swallowing, are needed to confirm our findings.

## Data Availability

Clinical and MRI data used to support the findings of this study are restricted by the Surrey Borders research ethics committee in order to protect patient privacy. Data are available from Dr Flavia Niccolini, Maurice Wohl Clinical Neuroscience Institute Institute of Psychiatry, Psychology & Neuroscience, King’s College London, 125 Coldharbour Lane, Camberwell, London SE5 9NU, UK, email: flavia.niccolini@kcl.ac.uk, 0044-(0)-207-848-5755 for researchers who meet the criteria for access to confidential data.

## Abbreviations

LED: levodopa equivalent dose
MRI: Magnetic Resonance Imaging
NMSQ: Non-motor Symptom Questionnaire
NMSS: Non-motor Symptom Scale
PDQ-39: 39-item Parkinson’s disease Questionnaire
